# Supplementary material for: Evaluation of antibody kinetics and durability in healthy individuals vaccinated with inactivated COVID-19 vaccine (CoronaVac): A cross-sectional and cohort study in Zhejiang, China
Source: eLife. 2023 Mar 16;12:e84056. doi: 10.7554/eLife.84056 (PMC10110235; doi:10.7554/eLife.84056)
Supplement: Supplementary file 1. — Table S1 Baseline characteristics for the cross-sectional survey; Table S2 Correspondence of neutralized fraction (%) and serum dilution; Table S3 The influence of sex, BMI, and chronic condition to GMT and Seropositivity. [file elife-84056-supp1.docx]

**Supplementary Tables**

**Table S1** Baseline characteristics for the cross-sectional survey.

|  | N=1067 | P (%) | |
| --- | --- | --- | --- |
| ﻿Age group (years) | | |  |
| 18-29 | 188 | 17.6 | |
| 30-39 | 340 | 31.9 | |
| 40-49 | 312 | 29.2 | |
| 50-59 | 227 | 21.3 | |
| Sex |  |  | |
| Male | 462 | 43.3 | |
| Female | 605 | 56.7 | |

**Table S2** Correspondence of neutralized fraction (%) and serum dilution.

| Neutralized fraction (%)  Serum dilution | WT | | Delta | | Omicron | |
| --- | --- | --- | --- | --- | --- | --- |
|  | P1 | P2 | P1 | P2 | P1 | P2 |
| 1:100 | 98.19 | 98.68 | 88.16 | 91.74 | 59.9 | 50.14 |
| 1:200 | 92.77 | 97.74 | 67.88 | 68.85 | 24.86 | 0 |
| 1:400 | 78.31 | 92.28 | 50.47 | 37.7 | 6.63 | 0 |
| 1:800 | 55.62 | 73.06 | 24.81 | 15.52 | 0 | 0 |
| 1:1600 | 29.52 | 48.94 | 5.43 | 0 | 0 | 0 |
| 1:3200 | 18.07 | 41.97 | 0 | 12.92 | 0 | 0 |
| 1:6400 | 6.83 | 25.2 | 3.1 | 0 | 10.89 | 0 |

P1 and P2 means serum sample of person1 and person2. The samples were conducted with the serial dilution from 1:100 and tested with pseudovirus-based neutralization.

**Table S3** The influence of sex, BMI, and chronic condition to GMT and Seropositivity.

| ﻿Time point | ﻿Statistic | Sex | | P | BMI (kg/m2) | | | P | Chronic conditions | | P |
| --- | --- | --- | --- | --- | --- | --- | --- | --- | --- | --- | --- |
|  |  | Male | ﻿Female |  | < 18.5 | 18.5-23.9 | ≥24 |  | Yes | No |  |
| Pre-V | ﻿GMT | 2.0 | 2.2 | 0.251 | 2.0 | 2.2 | 2.0 | 0.694 | 2.1 | 2.1 | 0.528 |
|  | ﻿95% CI | 1.96-2.1 | 2.0-2.4 |  | 2.2-2.0 | 2.0-2.3 | 2.0-2.1 |  | 1.9-2.2 | 2.0-2.3 |  |
|  | ﻿Seropositivity (%) | 0.0 | 6 |  | 0.0 | 5.3 | 0.0 |  | 3.3 | 3.3 |  |
| V2-1m | ﻿GMT | 27.6 | 31.0 | 0.534 | 16.6 | 32.4 | 26.0 | 0.137 | 24.9 | 32.0 | 0.134 |
|  | ﻿95% CI | 22.3-34.1 | 24.9-38.7 |  | 7.0-39.5 | 27.1-38.7 | 19.2-35.3 |  | 18.9-32.9 | 26.6-38.4 |  |
|  | ﻿Seropositivity (%) | 2.5 | 100.0 |  | 100.0 | 100.0 | 96.7 |  | 96.7 | 100.0 |  |
| V2-3m | ﻿GMT | 13.9 | 16.9 | 0.371 | 20.2 | 15.6 | 14.8 | 0.686 | 12.0 | 17.5 | 0.051 |
|  | ﻿95% CI | 10.6-18.1 | 14.2-20.3 |  | 2.8-147.2 | 13.0-18.8 | 11.0-20.0 |  | 9.2-15.8 | 14.6-21.0 |  |
|  | ﻿Seropositivity (%) | 90 | 100.0 |  | 100.0 | 96.5 | 93.3 |  | 93.3 | 96.7 |  |
| V2-6m | ﻿GMT | 5.8 | 7.3 | 0.233 | 5.8 | 6.5 | 6.8 | 0.967 | 5.6 | 7.2 | 0.231 |
|  | ﻿95% CI | 4.4-7.6 | 5.5-9.7 |  | 0.6-59.5 | 5.1-8.3 | 4.6-9.9 |  | 4.1-7.6 | 5.5-9.2 |  |
|  | ﻿Seropositivity (%) | 50 | 64 |  | 66.7 | 54.4 | 60.0 |  | 56.7 | 60.0 |  |
| V3-1m | ﻿GMT | 136.1 | 199.3 | 0.039 | 146.5 | 165.8 | 175.3 |  | 145.1 | 181.1 | 0.229 |
|  | ﻿95% CI | 99.9-185.4 | 159.2-249.5 |  | 81.9-262.1 | 130.2-211.1 | 125.2-245.5 | 0.862 | 107.0-196.9 | 142.8-229.7 |  |
|  | ﻿Seropositivity (%) | 100.0 | 100.0 |  | 100.0 | 100.0 | 100.0 |  | 100.0 | 100.0 |  |

GMT, geometric mean titers. The seropositivity rate is when positive NT50 is above 1:4.
