# Supplementary material for: Evaluation of antibody kinetics and durability in healthy individuals vaccinated with inactivated COVID-19 vaccine (CoronaVac): A cross-sectional and cohort study in Zhejiang, China
Source: eLife. 2023 Mar 16;12:e84056. doi: 10.7554/eLife.84056 (PMC10110235; doi:10.7554/eLife.84056)
Supplement: Supplementary file 2. [file elife-84056-supp2.docx]

## Statistical analysis

**﻿****Sample size determination**

No explicit power analysis was used in this study; sample size was determined on the maximum number of people recruited in research sites.

**Figure2**

Dates are presented as mean ± SEM. Neutralizing antibodies are presented as geometric mean titers (GMT), and their 95% confidence interval (CI) was calculated with Student’s t distribution on log-transformed data and then back-transformed. One-way analysis of variance was used for comparison. Correlations were assessed using Pearson’s correlation coefficient. Two-tailed P values were calculated. ns, not significant, * P< 0.05, **P < 0.01, ***P < 0.001.

| IgM-S+N | Pre-V | V1-14d | V1-28d | V2-1m | V2-3m | V2-6m | V2-9m | V2-12m |
| --- | --- | --- | --- | --- | --- | --- | --- | --- |
| Sample number | 91 | 125 | 91 | 100 | 110 | 160 | 190 | 200 |
| Mean | 0.4 | 5.1 | 5.1 | 1.4 | 0.4 | 0.3 | 0.3 | 0.2 |
| Std. Deviation | 0.2 | 11.7 | 9.1 | 2.4 | 0.7 | 0.5 | 0.5 | 0.4 |
| Std. Error of Mean | 0.0 | 1.1 | 0.9 | 0.2 | 0.1 | 0.04 | 0.04 | 0.03 |
| positive number | 3 | 72 | 69 | 33 | 4 | 7 | 9 | 4 |
| positive rate% | 3.3 | 57.6 | 75.8 | 33.0 | 3.6 | 4.4 | 4.7 | 2.0 |
|  |  |  |  |  |  |  |  |  |

| Multiple comparisons test | Below threshold? | Summary | Adjusted P Value |  |
| --- | --- | --- | --- | --- |
| Pre-V vs. V1-14d | Yes | **** | <0.0001 |  |
| Pre-V vs. V1-28d | Yes | **** | <0.0001 |  |
| Pre-V vs. V2-1m | No | ns | 0.8517 |  |
| Pre-V vs. V2-3m | No | ns | >0.9999 |  |
| Pre-V vs. V2-6m | No | ns | >0.9999 |  |
| Pre-V vs. V2-9m | No | ns | >0.9999 |  |
| Pre-V vs. V2-12m | No | ns | >0.9999 |  |
| V1-14d vs. V1-28d | No | ns | >0.9999 |  |
| V1-14d vs. V2-1m | Yes | **** | <0.0001 |  |
| V1-14d vs. V2-3m | Yes | **** | <0.0001 |  |
| V1-14d vs. V2-6m | Yes | **** | <0.0001 |  |
| V1-14d vs. V2-9m | Yes | **** | <0.0001 |  |
| V1-14d vs. V2-12m | Yes | **** | <0.0001 |  |
| V1-28d vs. V2-1m | Yes | **** | <0.0001 |  |
| V1-28d vs. V2-3m | Yes | **** | <0.0001 |  |
| V1-28d vs. V2-6m | Yes | **** | <0.0001 |  |
| V1-28d vs. V2-9m | Yes | **** | <0.0001 |  |
| V1-28d vs. V2-12m | Yes | **** | <0.0001 |  |
| V2-1m vs. V2-3m | No | ns | 0.8149 |  |
| V2-1m vs. V2-6m | No | ns | 0.6564 |  |
| V2-1m vs. V2-9m | No | ns | 0.6368 |  |
| V2-1m vs. V2-12m | No | ns | 0.4681 | |
| V2-3m vs. V2-6m | No | ns | >0.9999 | |
| V2-3m vs. V2-9m | No | ns | >0.9999 | |
| V2-3m vs. V2-12m | No | ns | >0.9999 | |
| V2-6m vs. V2-9m | No | ns | >0.9999 | |
| V2-6m vs. V2-12m | No | ns | >0.9999 | |
| V2-9m vs. V2-12m | No | ns | >0.9999 | |
|  |  |  |  | |

| IgG-S+N | Pre-V | V1-14d | V1-28d | V2-1m | V2-3m | V2-6m | V2-9m | V2-12m |
| --- | --- | --- | --- | --- | --- | --- | --- | --- |
| Sample number | 91 | 125 | 91 | 100 | 110 | 160 | 190 | 200 |
| Mean | 0.6 | 3.7 | 64.3 | 79.7 | 29.4 | 10.5 | 8.9 | 6.8 |
| Std. Deviation | 0.6 | 5.6 | 55.3 | 57.1 | 24.8 | 11.3 | 14.1 | 13.1 |
| Std. Error of Mean | 0.1 | 0.5 | 5.8 | 5.7 | 2.4 | 0.9 | 1.0 | 0.9 |
| positive number | 0 | 9 | 89 | 97 | 97 | 52 | 42 | 27 |
| positive rate% | 0.0 | 7.2 | 97.8 | 97.0 | 88.2 | 32.5 | 22.1 | 13.5 |

| Multiple comparisons test | Below threshold? | Summary | Adjusted P Value |
| --- | --- | --- | --- |
| Pre-V vs. V1-14d | No | ns | 0.9912 |
| Pre-V vs. V1-28d | Yes | **** | <0.0001 |
| Pre-V vs. V2-1m | Yes | **** | <0.0001 |
| Pre-V vs. V2-3m | Yes | **** | <0.0001 |
| Pre-V vs. V2-6m | No | ns | 0.0936 |
| Pre-V vs. V2-9m | No | ns | 0.2236 |
| Pre-V vs. V2-12m | No | ns | 0.5955 |
| V1-14d vs. V1-28d | Yes | **** | <0.0001 |
| V1-14d vs. V2-1m | Yes | **** | <0.0001 |
| V1-14d vs. V2-3m | Yes | **** | <0.0001 |
| V1-14d vs. V2-6m | No | ns | 0.3971 |
| V1-14d vs. V2-9m | No | ns | 0.6856 |
| V1-14d vs. V2-12m | No | ns | 0.9701 |
| V1-28d vs. V2-1m | Yes | ** | 0.0020 |
| V1-28d vs. V2-3m | Yes | **** | <0.0001 |
| V1-28d vs. V2-6m | Yes | **** | <0.0001 |
| V1-28d vs. V2-9m | Yes | **** | <0.0001 |
| V1-28d vs. V2-12m | Yes | **** | <0.0001 |
| V2-1m vs. V2-3m | Yes | **** | <0.0001 |
| V2-1m vs. V2-6m | Yes | **** | <0.0001 |
| V2-1m vs. V2-9m | Yes | **** | <0.0001 |
| V2-1m vs. V2-12m | Yes | **** | <0.0001 |
| V2-3m vs. V2-6m | Yes | **** | <0.0001 |
| V2-3m vs. V2-9m | Yes | **** | <0.0001 |
| V2-3m vs. V2-12m | Yes | **** | <0.0001 |
| V2-6m vs. V2-9m | No | ns | 0.9994 |
| V2-6m vs. V2-12m | No | ns | 0.9028 |
| V2-9m vs. V2-12m | No | ns | 0.9942 |

| IgM-RBD | Pre-V | V2-1m | V2-3m | V2-6m | V2-9m | V2-12m |
| --- | --- | --- | --- | --- | --- | --- |
| Sample number |  | 100 | 110 | 160 | 190 | 200 |
| Mean | 0.1 | 1.8 | 0.5 | 0.6 | 0.7 | 0.1 |
| Std. Deviation | 0.1 | 4.5 | 0.4 | 0.7 | 1.0 | 0.3 |
| Std. Error of Mean | 0.01 | 0.4 | 0.04 | 0.1 | 0.1 | 0.02 |
| positive number | 0 | 38.0 | 10.0 | 34.0 | 65.0 | 3.0 |
| positive rate% |  | 38.0 | 9.1 | 21.3 | 34.2 | 1.5 |

| Multiple comparisons test | Below threshold? | Summary | Adjusted P Value |
| --- | --- | --- | --- |
| Pre-V vs. V2-1m | Yes | **** | <0.0001 |
| Pre-V vs. V2-3m | No | ns | 0.5111 |
| Pre-V vs. V2-6m | No | ns | 0.2120 |
| Pre-V vs. V2-9m | No | ns | 0.0711 |
| Pre-V vs. V2-12m | No | ns | 0.9997 |
| V2-1m vs. V2-3m | Yes | **** | <0.0001 |
| V2-1m vs. V2-6m | Yes | **** | <0.0001 |
| V2-1m vs. V2-9m | Yes | **** | <0.0001 |
| V2-1m vs. V2-12m | Yes | **** | <0.0001 |
| V2-3m vs. V2-6m | No | ns | 0.9977 |
| V2-3m vs. V2-9m | No | ns | 0.9411 |
| V2-3m vs. V2-12m | No | ns | 0.4531 |
| V2-6m vs. V2-9m | No | ns | 0.9958 |
| V2-6m vs. V2-12m | No | ns | 0.1078 |
| V2-9m vs. V2-12m | Yes | * | 0.0166 |

| IgG-RBD | Pre-V | V2-1m | V2-3m | V2-6m | V2-9m | V2-12m |
| --- | --- | --- | --- | --- | --- | --- |
| Sample number |  | 100 | 110 | 160 | 190 | 200 |
| Mean | 0.04 | 18.0 | 5.6 | 2.3 | 2.1 | 1.9 |
| Std. Deviation | 0.08 | 16.3 | 4.8 | 2.0 | 3.2 | 3.9 |
| Std. Error of Mean | 0.01 | 1.6 | 0.5 | 0.2 | 0.2 | 0.3 |
| positive number | 0.00 | 97.0 | 110.0 | 121.0 | 119.0 | 93.0 |
| positive rate% |  | 97.0 | 100.0 | 75.6 | 62.6 | 46.5 |

| Multiple comparisons test | Below threshold? | Summary | Adjusted P Value |
| --- | --- | --- | --- |
| Pre-V vs. V2-1m | Yes | **** | <0.0001 |
| Pre-V vs. V2-3m | Yes | **** | <0.0001 |
| Pre-V vs. V2-6m | No | ns | 0.1086 |
| Pre-V vs. V2-9m | No | ns | 0.1748 |
| Pre-V vs. V2-12m | No | ns | 0.2370 |
| V2-1m vs. V2-3m | Yes | **** | <0.0001 |
| V2-1m vs. V2-6m | Yes | **** | <0.0001 |
| V2-1m vs. V2-9m | Yes | **** | <0.0001 |
| V2-1m vs. V2-12m | Yes | **** | <0.0001 |
| V2-3m vs. V2-6m | Yes | *** | 0.0006 |
| V2-3m vs. V2-9m | Yes | **** | <0.0001 |
| V2-3m vs. V2-12m | Yes | **** | <0.0001 |
| V2-6m vs. V2-9m | No | ns | 0.9993 |
| V2-6m vs. V2-12m | No | ns | 0.9935 |
| V2-9m vs. V2-12m | No | ns | >0.9999 |

| pseudovirus | Pre-V | V2-1m | V2-3m | V2-6m | V2-9m | V2-12m |
| --- | --- | --- | --- | --- | --- | --- |
| Sample number |  | 100 | 110 | 160 | 190 | 200 |
| Mean | 3.2 | 76.2 | 55.7 | 37.5 | 26.6 | 24.0 |
| Std. Deviation | 6.2 | 16.2 | 17.3 | 18.8 | 18.5 | 21.7 |
| Std. Error of Mean | 0.7 | 1.6 | 1.7 | 1.5 | 1.3 | 1.5 |
| positive number | 0.00 | 92.0 | 68.0 | 40.0 | 22.0 | 23.0 |
| positive rate% |  | 92.0 | 61.8 | 25.0 | 11.6 | 11.5 |

| Multiple comparisons test | Below threshold? | Summary | Adjusted P Value |
| --- | --- | --- | --- |
| Pre-V vs. V-1m | Yes | **** | <0.0001 |
| Pre-V vs. V-3m | Yes | **** | <0.0001 |
| Pre-V vs. V-6m | Yes | **** | <0.0001 |
| Pre-V vs. V-9m | Yes | **** | <0.0001 |
| Pre-V vs. V-12m | Yes | **** | <0.0001 |
| V-1m vs. V-3m | Yes | **** | <0.0001 |
| V-1m vs. V-6m | Yes | **** | <0.0001 |
| V-1m vs. V-9m | Yes | **** | <0.0001 |
| V-1m vs. V-12m | Yes | **** | <0.0001 |
| V-3m vs. V-6m | Yes | **** | <0.0001 |
| V-3m vs. V-9m | Yes | **** | <0.0001 |
| V-3m vs. V-12m | Yes | **** | <0.0001 |
| V-6m vs. V-9m | Yes | **** | <0.0001 |
| V-6m vs. V-12m | Yes | **** | <0.0001 |
| V-9m vs. V-12m | No | ns | 0.7256 |

| Neutralizing antibody | Pre-V | V2-1m | V2-3m | V2-6m | V2-9m | V2-12m |
| --- | --- | --- | --- | --- | --- | --- |
| Sample number |  | 100 | 110 | 160 | 190 | 200 |
| GMT | 2.2 | 20.2 | 14.7 | 7.8 | 6.0 | 4.1 |
| Lower 95% CI of geo. mean | 2.1 | 16.3 | 12.7 | 6.9 | 5.3 | 3.7 |
| Upper 95% CI of geo. mean | 2.3 | 24.9 | 17.0 | 8.8 | 6.7 | 4.5 |
| 25% Percentile | 2 | 12 | 8 | 6 | 4 | 2 |
| 75% Percentile | 2 | 32 | 24 | 15 | 8 | 6 |

| Multiple comparisons test | Below threshold? | Summary | Adjusted P Value |
| --- | --- | --- | --- |
| Pre-V vs. V-1m | Yes | **** | <0.0001 |
| Pre-V vs. V-3m | Yes | **** | <0.0001 |
| Pre-V vs. V-6m | Yes | **** | <0.0001 |
| Pre-V vs. V-9m | Yes | **** | <0.0001 |
| Pre-V vs. V-12m | Yes | **** | <0.0001 |
| V-1m vs. V-3m | No | ns | 0.1872 |
| V-1m vs. V-6m | Yes | **** | <0.0001 |
| V-1m vs. V-9m | Yes | **** | <0.0001 |
| V-1m vs. V-12m | Yes | **** | <0.0001 |
| V-3m vs. V-6m | Yes | **** | <0.0001 |
| V-3m vs. V-9m | Yes | **** | <0.0001 |
| V-3m vs. V-12m | Yes | **** | <0.0001 |
| V-6m vs. V-9m | Yes | * | 0.0239 |
| V-6m vs. V-12m | Yes | **** | <0.0001 |
| V-9m vs. V-12m | Yes | **** | <0.0001 |

**Figure3**

Dates are presented as mean ± SEM. Neutralizing antibodies are presented as geometric mean titers (GMT), and their 95% confidence interval (CI) was calculated with Student’s t distribution on log-transformed data and then back-transformed. One-way analysis of variance was used for comparison. Correlations were assessed using Pearson’s correlation coefficient. Two-tailed P values were calculated. ns, not significant, * P< 0.05, **P < 0.01, ***P < 0.001.

| IgG-RBD | V2-1m | V2-3m | V2-6m | V3-1m |
| --- | --- | --- | --- | --- |
| Sample number | 90.0 | 90.0 | 90.0 | 90.0 |
| Mean | 9.4 | 2.8 | 2.8 | 21.7 |
| Std. Deviation | 7.9 | 3.0 | 2.4 | 16.8 |
| Std. Error of Mean | 0.8 | 0.3 | 0.2 | 1.8 |
| positive number | 90.0 | 74.0 | 78.0 | 89.0 |
| positive rate% | 100.0 | 82.2 | 86.7 | 98.9 |

| Multiple comparisons test | Below threshold? | Summary | Adjusted P Value |
| --- | --- | --- | --- |
| Pre-V vs. V2-1m | Yes | **** | <0.0001 |
| Pre-V vs. V2-3m | Yes | **** | <0.0001 |
| Pre-V vs. V2-6m | Yes | **** | <0.0001 |
| Pre-V vs. V3-1m | Yes | **** | <0.0001 |
| V2-1m vs. V2-3m | Yes | **** | <0.0001 |
| V2-1m vs. V2-6m | Yes | **** | <0.0001 |
| V2-1m vs. V3-1m | Yes | **** | <0.0001 |
| V2-3m vs. V2-6m | No | ns | >0.9999 |
| V2-3m vs. V3-1m | Yes | **** | <0.0001 |
| V2-6m vs. V3-1m | Yes | **** | <0.0001 |

| IgG-S+N | V2-1m | V2-3m | V2-6m | V3-1m |
| --- | --- | --- | --- | --- |
| Sample number | 90.0 | 90.0 | 90.0 | 90.0 |
| Mean | 67.4 | 24.3 | 9.4 | 131.3 |
| Std. Deviation | 47.2 | 23.0 | 15.1 | 81.1 |
| Std. Error of Mean | 5.0 | 2.5 | 1.6 | 8.6 |
| positive number | 87.0 | 66.0 | 28.0 | 89.0 |
| positive rate% | 96.7 | 73.3 | 31.1 | 98.9 |

| Multiple comparisons test | Below threshold? | Summary | Adjusted P Value |
| --- | --- | --- | --- |
| Pre-V vs. V2-1m | Yes | **** | <0.0001 |
| Pre-V vs. V2-3m | Yes | **** | <0.0001 |
| Pre-V vs. V2-6m | Yes | **** | <0.0001 |
| Pre-V vs. V3-1m | Yes | **** | <0.0001 |
| V2-1m vs. V2-3m | Yes | **** | <0.0001 |
| V2-1m vs. V2-6m | Yes | **** | <0.0001 |
| V2-1m vs. V3-1m | Yes | **** | <0.0001 |
| V2-3m vs. V2-6m | Yes | **** | <0.0001 |
| V2-3m vs. V3-1m | Yes | **** | <0.0001 |
| V2-6m vs. V3-1m | Yes | **** | <0.0001 |

| Neutralizing antibody | Pre-V | V2-1m | V2-3m | V2-6m | V3-1m |
| --- | --- | --- | --- | --- | --- |
| Sample number | 90.0 | 89.0 | 88.0 | 90.0 | 90.0 |
| Geometric mean | 2.1 | 29.4 | 15.5 | 6.6 | 168.2 |
| Geometric SD factor | 1.3 | 2.1 | 2.1 | 2.6 | 2.4 |
| Lower 95% CI of geo. mean | 2.0 | 25.3 | 13.3 | 5.4 | 139.7 |
| Upper 95% CI of geo. mean | 2.2 | 34.2 | 18.0 | 8.0 | 202.6 |
| positive number | 3.0 | 88.0 | 84.0 | 52.0 | 90.0 |
| positive rate% | 3.3 | 98.9 | 95.5 | 57.8 | 100.0 |

| Multiple comparisons test | Below threshold? | Summary | Adjusted P Value |
| --- | --- | --- | --- |
| Pre-V vs. V2-1m | Yes | **** | <0.0001 |
| Pre-V vs. V2-3m | Yes | **** | <0.0001 |
| Pre-V vs. V2-6m | Yes | **** | <0.0001 |
| Pre-V vs. V3-1m | Yes | **** | <0.0001 |
| V2-1m vs. V2-3m | Yes | **** | <0.0001 |
| V2-1m vs. V2-6m | Yes | **** | <0.0001 |
| V2-1m vs. V3-1m | Yes | **** | <0.0001 |
| V2-3m vs. V2-6m | Yes | **** | <0.0001 |
| V2-3m vs. V3-1m | Yes | **** | <0.0001 |
| V2-6m vs. V3-1m | Yes | **** | <0.0001 |

| pseudovirus | Pre-V | V2-1m | V2-3m | V2-6m | V3-1m |
| --- | --- | --- | --- | --- | --- |
| Sample number | 90.0 | 89.0 | 88.0 | 89.0 | 90.0 |
| Mean | 8.6 | 72.7 | 44.1 | 21.4 | 84.3 |
| Std. Deviation | 9.4 | 14.7 | 16.6 | 16.4 | 16.2 |
| Std. Error of Mean | 1.0 | 1.6 | 1.8 | 1.7 | 1.7 |
| positive number | 0.0 | 80.0 | 33.0 | 6.0 | 86.0 |
| positive rate% | 0.0 | 89.9 | 37.5 | 6.7 | 95.6 |

| Multiple comparisons test | Below threshold? | Summary | Adjusted P Value |
| --- | --- | --- | --- |
| Pre-V vs. V2-1m | Yes | **** | <0.0001 |
| Pre-V vs. V2-3m | Yes | **** | <0.0001 |
| Pre-V vs. V2-6m | Yes | **** | <0.0001 |
| Pre-V vs. V3-1m | Yes | **** | <0.0001 |
| V2-1m vs. V2-3m | Yes | **** | <0.0001 |
| V2-1m vs. V2-6m | Yes | **** | <0.0001 |
| V2-1m vs. V3-1m | Yes | **** | <0.0001 |
| V2-3m vs. V2-6m | Yes | **** | <0.0001 |
| V2-3m vs. V3-1m | Yes | **** | <0.0001 |
| V2-6m vs. V3-1m | Yes | **** | <0.0001 |

**Figure4**

Dates are presented as mean ± SEM. Neutralizing antibodies are presented as geometric mean titers (GMT), and their 95% confidence interval (CI) was calculated with Student’s t distribution on log-transformed data and then back-transformed. The paired Student's t-test and one-way analysis of variance were used for comparison. Two-tailed P values were calculated. ns, not significant, * P< 0.05, **P < 0.01, ***P < 0.001.

**V2-1m**

| Multiple comparisons test | Below threshold? | Summary | Adjusted P Value |
| --- | --- | --- | --- |
| WT vs. Delta | Yes | **** | <0.0001 |
| WT vs. Omicron | Yes | **** | <0.0001 |
| Delta vs. Omicron | Yes | **** | <0.0001 |

**V3-1m**

| Tukey's multiple comparisons test | Below threshold? | Summary | Adjusted P Value |
| --- | --- | --- | --- |
| WT vs. Delta | Yes | **** | <0.0001 |
| WT vs. Omicron | Yes | **** | <0.0001 |
| Delta vs. Omicron | Yes | **** | <0.0001 |

**Delta**

| Paired t test |  |
| --- | --- |
| P value | 0.0268 |
| P value summary | * |
| Significantly different (P < 0.05)? | Yes |
| One- or two-tailed P value? | Two-tailed |
| t, df | t=2.253, df=88 |
| Number of pairs | 89 |

**Omicron**

| Paired t test |  |
| --- | --- |
| P value | 0.0035 |
| P value summary | ** |
| Significantly different (P < 0.05)? | Yes |
| One- or two-tailed P value? | Two-tailed |
| t, df | t=3.002, df=88 |
| Number of pairs | 89 |
